# Physiological roles and therapeutic implications of USP6

**DOI:** 10.1038/s41420-025-02466-0

**Published:** 2025-05-10

**Authors:** Suaad Syed, Muhammad Yasir Khan Painda, Dawood Ghafoor, Dongjin Gu, Feng Wang

**Affiliations:** 1 Key Laboratory of Molecular Medicine and Biotherapy, School of Life Science, Beijing Institute of Technology, Beijing, 100081 China; 2 School of Chemistry and Chemical Engineering, Beijing Institute of Technology, Beijing, 100081 China; 3 Veterinary Preclinical Sciences, College of Science and Engineering (CSE), James Cook University, Townsville, QLD 4811 Australia

**Keywords:** Oncogene proteins, Ubiquitins

## Abstract

Ubiquitin-specific protease 6 (USP6) is a member of deubiquitinating enzyme family, recognized for its essential roles in physiological and pathological processes. USP6 is initially identified as a hominoid-specific enzyme residing on chromosome 17p13. USP6 is involved in regulating cellular functions, signaling pathways, protein degradation, intracellular trafficking, tumorigenesis and immune responses. USP6 is pivotal in signaling pathways, including NF-κB, JAK-STAT, and Wnt, which are fundamental for maintaining cellular homeostasis and mediating stress responses. Dysregulation of USP6 has been implicated in a spectrum of diseases, including bone tumors, breast and colorectal cancers, cranial fasciitis, and neurological disorders such as memory dysfunction. Furthermore, USP6 is involved in emerging therapeutic strategies highlighting its implications for drug development. A number of potential small molecule inhibitors are known to be responsible for suppression of USP6, such as Momelotinib (CYT387), FT385, USP30 Inh-1, -2 and -3, 2,6-Diaminopyridine-3,5-bis(thiocyanate) (PR-619) and so on. This review explores the emerging role of USP6 as a key regulator of cellular signaling pathways, its involvement in disease progression, its physiological functions, and the inhibitors that effectively suppress USP6 activity in detail. The comprehensive study provides insight to enhance our understanding of biological importance and therapeutic interventions of USP6 in drug development.

## Facts

- USP6 functions as a deubiquitinase and a designated oncogene, responsible for various cancers and sarcomas progression.
- USP6 modulates key pathways like NF-κB, JAK-STAT and Wnt/β-catenin driving tumor progression and inflammation.
- USP6 is implicated in a variety of cell biological processes, including cell cycle regulation, proliferation, invasion, migration, and DNA damage repair.

## Open Questions

- Can USP6 serve as a potential biomarker for early detection of cancers or sarcomas where it is overexpressed?
- What are the downstream effects of USP6 inhibition on tumor invasion and inflammation?
- What specific inhibitors can effectively target USP6 without off-target effects?

## Introduction

Ubiquitination, a type of histone modification, regulates cellular localization, proteasomal degradation of targeted proteins, and protein interactions [1–3]. Protein ubiquitination involves a chain reaction consisting of ubiquitin-conjugating enzymes, ubiquitin-activating enzymes, and ubiquitin-protein ligases [4–6]. In contrast, Deubiquination is responsible for the dynamic and reversible ubiquitin modification of proteins by identifying ubiquitinated proteins and cleaving the ubiquitin [7]. In general, a unique ubiquitin-related network that controls numerous aspects of cellular function is made up of ubiquitinases and deubiquitinases. It is a key regulator in the growth and spread of different tumors, such as colorectal cancer (CRC), lung cancer, breast cancer and liver cancer, etc. It is directly linked to several cellular biological processes. To date, more than 490 DUBs have been identified from seven subfamilies. These subfamilies include zinc-finger containing ubiquitin peptidase (ZUP1), ovarian tumor proteases (OTU), ubiquitin C-terminal hydrolases (UCH), Motif Interacting with Ub-containing novel DUB family (MINDY), and Jab1/Mov34/MPN^+^ protease (JAMM)(JAMM) family includes zinc-binding metalloproteases and Ubiquitin-specific proteases (USP) [2, 8, 9] Ubiquitin-specific proteases (USPs) is the largest family of DUBs, consisting of 58 identified members known to have multiple conserved domains and comparable catalytic sites [10]. USPs belong to the class of cysteine-dependent proteases, which function similarly to papain, another cysteine protease. The majority of USPs comprised of 350–400 amino acids, although the catalytic domain, which is regarded as highly conserved USP domain, is the most conserved domain across USPs that range between 300–800 amino acids [11]. USPs can have multiple accessory domains in addition to the catalytic domain. The ubiquitin-like (UBL) domain, zinc finger ubiquitin-binding (ZnF-UBP) domain, domains unique to USP (DUSP), ubiquitin-interacting motifs (UIM), and ubiquitin-associated (UBA) are some of the most characteristic domains discovered in USP family members. The substrate specificity is determined by the unique intersection of distinct domains that distinguishes each USP [12, 13].

**Fig. 1:**
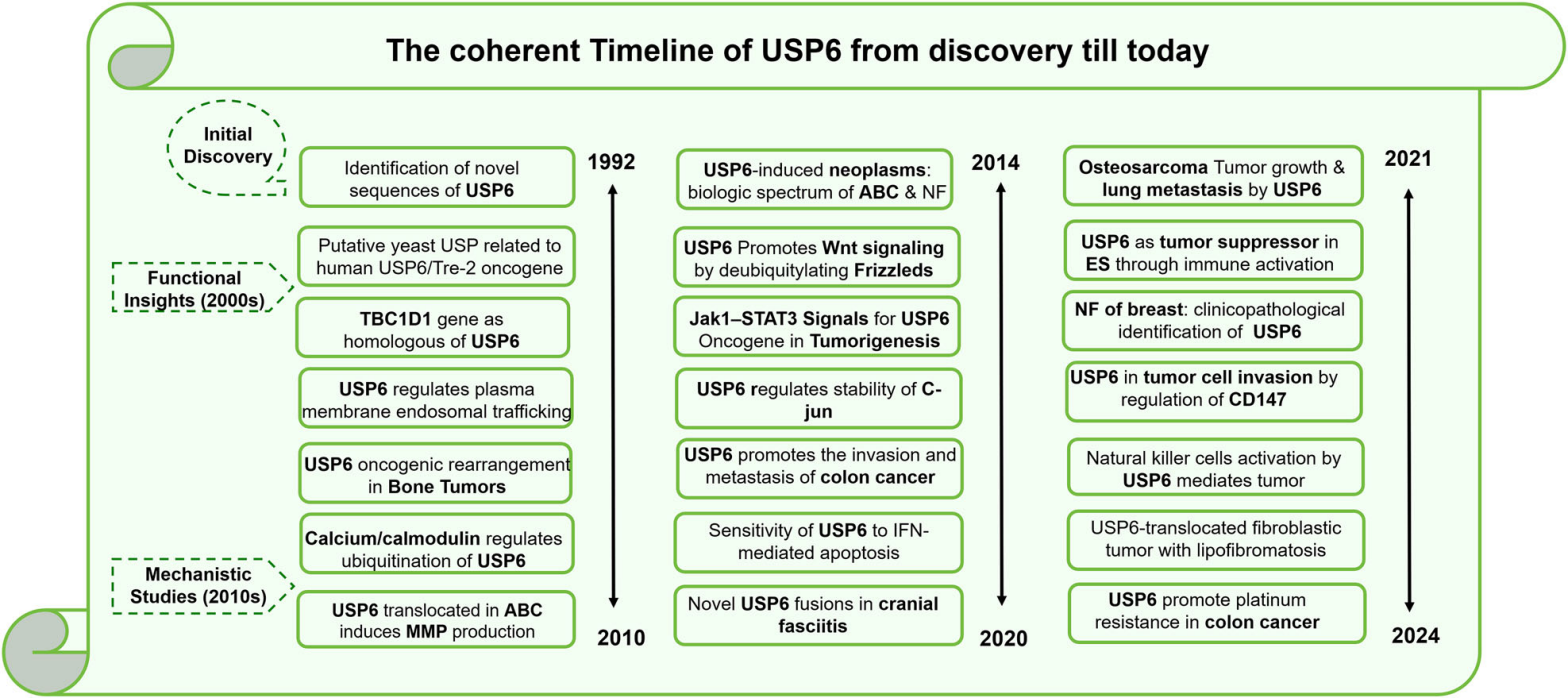
Research timeline of USP6 spanning from 1992 to 2024.

USPs control multiple cellular mechanisms which are crucial in cancer, such as chromatin remodeling, DNA damage repair mechanisms, signaling pathways, and cell cycle [14]. Mostly, USPs are considered to be associated indirectly or directly with cancer [15]. USPs are classified as cancer-associated proteases, and the creation of anti-cancer treatments has been the subject of multiple investigations [16]. USPs are considered prospective anti-cancer targets based on the comprehensive assessment of gene alterations and abnormal expression in different malignancies [17]. There are various types of ubiquitin-specific proteases (USPs), each contributing distinctively to tumorigenesis. Among these, USP6 has emerged as a critical regulator in the pathogenesis of diverse malignancies, including pancreatic cancer, aneurysmal bone cysts, breast cancer, nodular fasciitis, cranial fasciitis, colon cancer, and colorectal cancer. Recent evidence highlights its pivotal role in driving tumor progression and shaping the oncogenic microenvironment. Since USP6 was the first deubiquitinase to be identified as an oncogene, efforts have been made to learn more about the structural and physiological properties of this enzyme [18]. A research timeline of USP6 from its discovery until now is detailed in Fig. 1. USP6 was first discovered to be a transforming gene in oncogenic fusion proteins, sometimes referred to as USP6/TRE17 [19]. Due to tissue-specific expression, USP6 is also known as tre-2 [20]. In this review, we will discuss the structural features, physiochemical properties, physiological role, diseases associated with USP6 and inhibition of USP6 in detail. This review will provide a deep insight to understand the involvement of USP6 in various cellular pathways and possible knockdown procedures to discover its therapeutic potential.

## Ubiquitin-specific protease 6 (USP6)

USP6 is essential for several biological functions, such as survival, differentiation, and cell proliferation. It is expressed in the heart, brain, and skeletal muscles, among other tissues. Under normal circumstances, USP6 affects a number of cellular processes, including gene expression, apoptosis, cytoskeleton organization, and cell migration [21–24]. It also helps in maintaining protein homeostasis. Even though Ubiquitin-Specific Protease 6 (USP6)/TRE17 was first cloned twenty years ago, very little is known about its function in healthy or diseased organisms. By transfecting genomic DNA fragments from the Ewing sarcoma (ES) cell line IARC-EW1 into the mouse fibro-blast line NIH3T3, isolating transformants that triggered tumor growth, and xenografting into nude mice, USP6 was first discovered as a putative oncogene in 1992 [25–28].

**Fig. 2:**
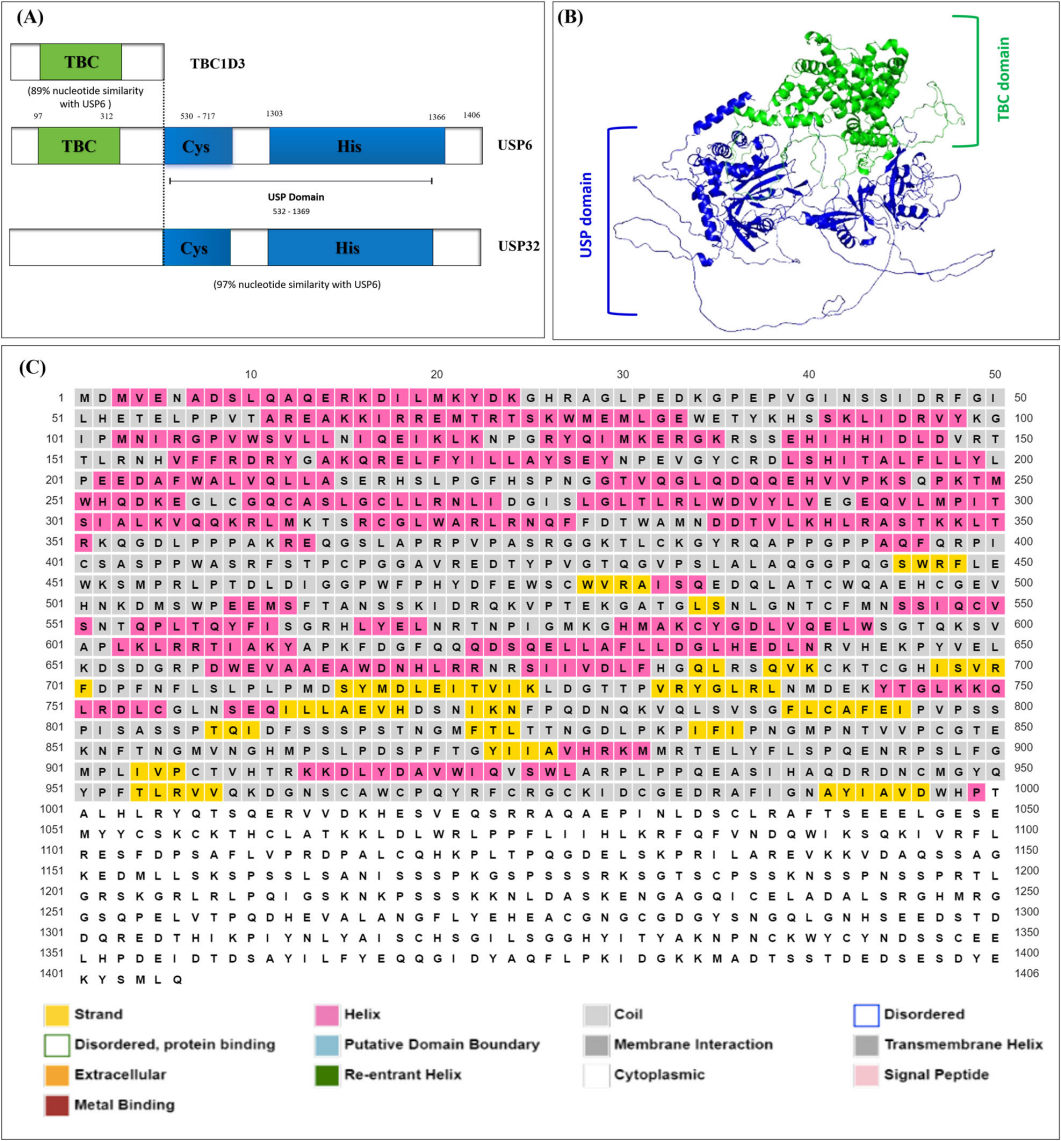
Simulated structure of USP6. **A** USP6 Domains and Isoforms USP6 comprised of 2 domains: USP domain and a Rap-GAP homology/TBC domain. The short isoform of USP6 is lacking histidine residue and thus has a non-functional USP domain. **B** Graphical presentation of secondary structure of protein obtained by AlphaFold 2, blue color indicating USP domain while green color representing the TBC domain. **C** The structure of full length human USP6 predicted by PSIPRED server, where Gray color show (>90 pLDDT), Pink (pLDDT > 70), and yellow (pLDDT > 50).

Over the following decade, USP6’s structure was analyzed, and it was found to have two primary domains: a functional ubiquitin-specific protease domain at the C-terminus and a TBC/Rab-GAP homology domain at the N-terminus **(**Fig. 2A**)**. USP6 encodes two principal splice variants: the full-length isoform (USP6) and a truncated variant, USP6(short), previously termed USP6 (onco) (Fig. 2A). The USP6(short) variant is characterized by a C-terminal truncation that results in the loss of a substantial portion of the TBC domain. In contrast, the full-length USP6 isoform retains the TBC/Rab-GAP homology and USP domains, which are integral to its functional architecture. Due to the truncation, a crucial histidine residue in the USP domain is absent, rendering USP6(short) ineffective as a deubiquitinase catalytically. Although TRE17/USP6/Tre-2 is one of the namesakes of this domain, it has turned out to be an atypical TBC protein, as it neither functions as a GAP nor targets a Rab. The domains of USP6 are highly similar to those of USP32 and TBC1D3, two other proteins. The USP domains of USP6 and USP32 have 97% identity, whereas the TBC domains of USP6 and TBC1D3 share about 80% amino acid identity [18].

Around 21–33 million years ago, during the advent of hominids, it was believed that duplications and subsequent translocations of USP32 and TBC1D3 gave rise to the USP6 gene [29]. Even though USP6, TBC1D3 and USP32 are homologs, USP6 has unique functionality [30, 31] of encoding GTPases of the Rab family by its TBC domains. The TBC domain of USP6 is catalytically inactive since it lacks several structural components, including a crucial arginine residue present in all other functioning TBC domains [32, 33]. Later on, it was found that TBC domain of USP6 interacts with Arf6, a GTPase member of the Arf family, rather than a Rab. It was shown that USP6 facilitates Arf6’s recruitment to the plasma membrane, where its guanine nucleotide exchange factors (GEF) are located, hence promoting GTP loading and Arf6 activation in vivo [34]. Since USP6’s secondary structure has not been experimentally confirmed, in this review we used the to predict the protein’s secondary structure to understand the characteristic features clearly using AlphaFold 2 [35–37]. The predicted structure revealed that the protein contains 20.0% coil structures, 49.1% a-helices, and 30.9% beta strands. The three-dimensional (3-D) structure of USP6 indicated two different color combinations, blue color indicating USP domain while green color representing the TBC domain (Fig. 2B). The structure of full-length USP6 predicted by PSIPRED server is given in (Fig. 2C), where Gray color shows (>90 Predicated Local Distance Difference Test (pLDDT)), Pink (pLDDT > 70), and yellow (pLDDT > 50).

### Physiochemical properties

USP6 protein has 1406 amino acids with a molecular weight of 158658.12 Da, a theoretical PI (isoelectric point) (7.87), aliphatic index (72.77), positive charged residues (168), and negative charger residues (164).

## Role of USP6 in cellular pathways

USP6 is a deubiquitinating enzyme that is involved in immune responses, cellular trafficking, inflammatory response, cell signaling, cytoskeleton and extracellular matrix remodeling, DNA damage repair, protein homeostasis (proteostasis), cell proliferation, cell apoptosis, and signal transduction and tumorigenesis. By removing ubiquitin molecules from proteins and controlling their stability, activity, and localization, USP6 stimulates the development and division of cells by maintaining the stability of important cell cycle regulators. USP6 controls the cytoskeleton’s dynamics by deubiquitinating and activating transcription factors, affecting gene expression, cell shape, motility, and adhesion [38, 39]. The important cellular pathways are discussed below.

### Canonical NFκB

**Fig. 3:**
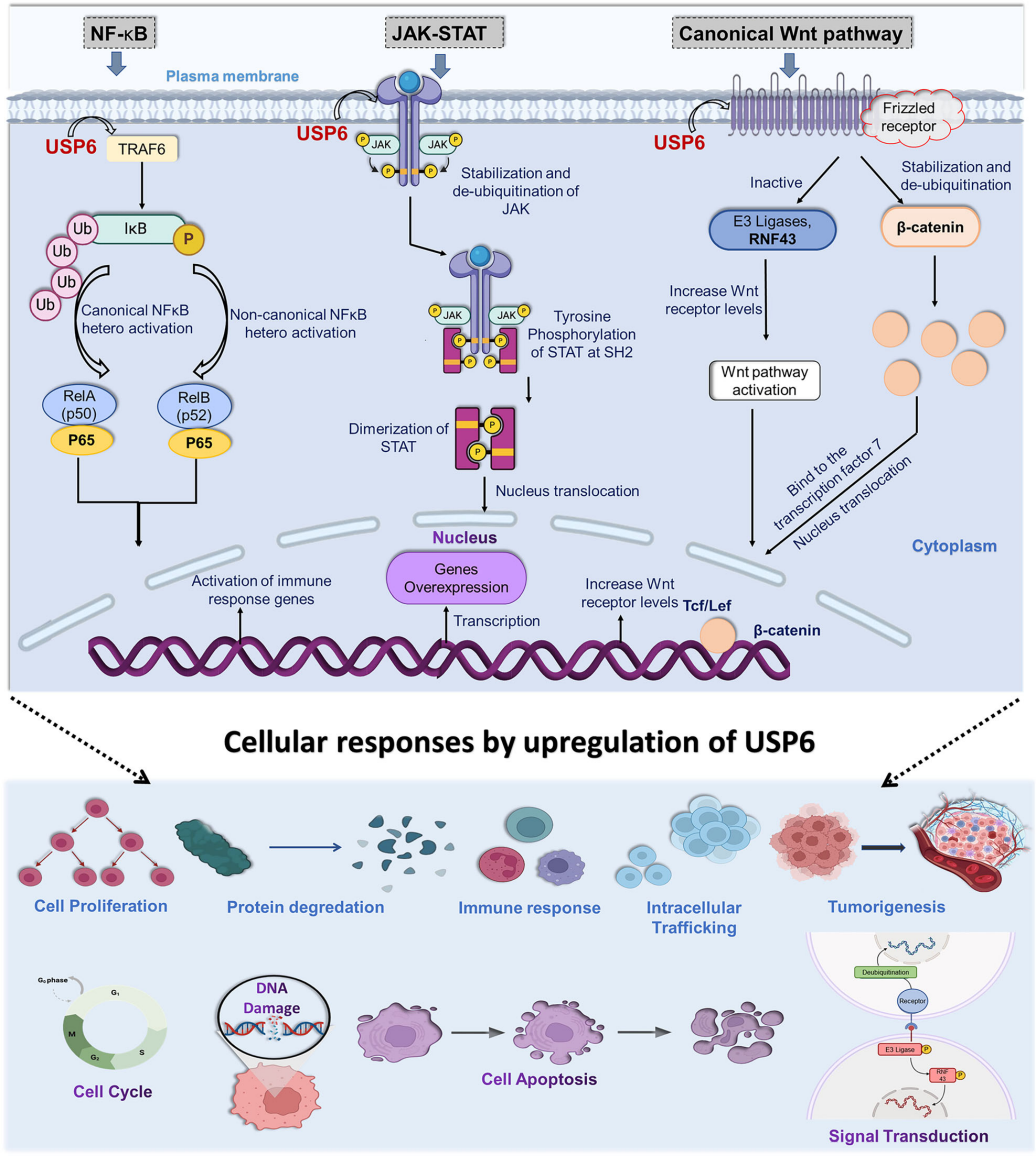
Molecular regulation and key biological processes of USP6, include hematopoiesis, DNA repair, inflammation, cell cycle progression, and embryogenesis.

The NFκB pathway, divided into canonical and noncanonical NFκB subsets, controls the transcription of several genes, including those involved in cytokines and apoptosis/survival factors [40–42]. A heterodimer of p65 and RelA (p50) or a p65 homodimer makes up the conventional NFκB transcription factor, while a heterodimer of p65 and RelB (p52) makes up the noncanonical factor. It was discovered that USP6 increased the canonical NFκB hetero- and homodimer’s nuclear accumulation and activation. Under typical circumstances, the activation of USP6 binds to IκB kinase (IKK complex). IKK is activated by USP6 binding to it, which then causes IKK to be phosphorylated and activates both canonical and noncanonical NFκB. In addition to promoting inflammation and stimulating immune cells, NFκB controls cell division and cell cycle advancement, as shown in Fig. 3 [43].

### JAK-STAT pathway

The cytoplasmic tyrosine kinases, the Janus kinase or JAK family (JAK1-JAK3), are traditionally linked to cytokine signaling. When cytokines bind to their specific receptors, JAK is recruited, phosphorylated, and activated. After JAK phosphorylation, JAK kinases target the tyrosine phosphorylation of a class of transcription factors called signal transducer and activator of transcription (STATs). STAT1-STAT6 are the seven members of the STAT family [44]. The signaling cascade of each receptor is normally regulated by one or two JAK members. Furthermore, every receptor-JAK combination attracts particular STATs, contributing to additional regulation of the signaling cascade. After being phosphorylated and activated, STATs can form homo- or hetero-dimers. Tyrosine phosphorylation is normally responsible for the activation and regulation of JAK family kinases [45]. Recently, however, it has been discovered that ubiquitination and proteasomal degradation play a crucial role in a unique regulatory mechanism that governs JAK. In vitro and in vivo, USP6 directly binds to and deubiquitinates JAK, protecting it from proteasomal destruction. Increased tyrosine phosphorylation and STAT activation were also caused by USP6’s stabilization and deubiquitination of JAK, as shown in Fig. 3 [46]. These pathways also control the expression of genes related to immune response, tumor growth, development, proliferation, survival of cells, differentiation, and other vital biological processes like haematopoiesis [4, 6, 46].

### Wnt pathway

Lately it has been discovered that USP6 stabilizes the Frizzled receptor and directly deubiquitinates it, activating the Wnt pathway [47]. The Wnt pathway, which is closely regulated at multiple stages, is essential for controlling the growth and homeostasis of many bodily tissues. Similar to NFκB, the Wnt pathway has two main routes: the β-catenin-dependent and the β-catenin-independent pathways.

The Wnt ligand binds to the Frizzled (Fzd) receptors, a family of 10 similar G protein-coupled receptors (referred to as Frizzled1–10), in the β-catenin–dependent pathway. Following Wnt binding, the destruction complex that normally targets β- catenin for degradation is inhibited, and β-catenin can subsequently accumulate, translocate to the nucleus, and bind to the TCF/LEF family of transcription factors to initiate transcription (Fig. 3).

USP6 sensitizes cells to Wnt signaling by targeting Fzds for deubiquitination, though only Fzd5 was shown to be directly deubiquitinated by USP6 [47]. Fzd receptors are targeted for ubiquitination and subsequent lysosomal degradation by the E3 ubiquitin ligases ZNRF3 and its homolog RNF43 [48]. Expression of USP6 in RNF43-inactive cells did not potentiate Wnt signaling, and reexpression of RNF43 restored USP6-enhanced Wnt signaling. Ablation of the USP domain significantly, but not completely disrupted Wnt activation. Arf6 activation has been demonstrated to contribute to classical Wnt signaling [49, 50]; consequently, the residual Wnt activation observed with a USP6 mutant lacking deubiquitinase activity may be attributable to Arf6-mediated signaling. However, this hypothesis has not yet been experimentally validated. The classical Wnt signaling pathway is integral to tissue homeostasis, cell differentiation, proliferation, regeneration, and embryonic development [48–50].

### Knockdown of USP6

Knocking down USP6 can be effectively achieved through techniques such as siRNA, shRNA, CRISPRi, ASOs, and modulation of miRNA. Each method offers unique advantages depending on the experimental context and desired duration of knockdown as discussed below;

Wnt signaling pathways have important roles in carcinogenesis. Michael et al. discovered that the deubiquitylase USP6 is a powerful stimulator of Wnt signaling. USP6 promotes Wnt signaling by deubiquitylating Fzds, increasing their cell-surface presence. In nodular fasciitis, chromosomal translocations cause overexpression of USP6, which activates the Wnt/β-catenin pathway [51].To further determine the role of Wnt signaling in USP6-mediated tumor growth, they tested USP6/NIH 3T3 cells with DKK1, a secreted protein that inhibits Fzd receptor activation. DKK1 significantly reduced USP6-induced β-catenin expression when compared to cells expressing USP6 alone. Xenografting these cells into NOG-SCID mice showed that DKK1 expression greatly suppressed USP6-dependent tumor growth. This suggests that Wnt signaling is a primary target of USP6 during carcinogenesis [47].

Developing effective therapy for bone and soft tissue tumors (BSTT) is challenging due to their lack of understanding. Chou et al. identify a crucial pathway for the USP6/TRE17 oncogene, which is overexpressed after chromosome translocation in a variety of human malignancies, including aneurysmal bone cyst (ABC) and its related benign lesion nodular fasciitis. The JAK1-STAT3 signaling pathway plays a crucial role in USP6-mediated BSTT production. To establish if the JAK1-STAT3 signaling axis is necessary for USP6-mediated transformation. JAK1 and STAT3 were removed from USP6/NIH3T3 using CRISPR-Cas9 genome editing. Clonal cell lines were created by deleting JAK1, STAT3, or both. Immunoblotting demonstrated that the JAK1 and STAT3 CRISPR constructs did not target other family members [46]. Cells lacking JAK1 (USP6/TRE17) showed no activation of STAT3 caused by USP6. Cells with monoallelic JAK1 deletion (USP6/TRE17) showed dramatically decreased STAT3 activation. JAK1 levels in this clone were similar to those in control NIH3T3 cells, indicating that USP6’s activation of STAT3 requires JAK1 overexpression. Xenografting CRISPR cells into immunodeficient mice indicated a crucial role for JAK1 and STAT3 in USP6-induced tumor development. Depleting JAK1 significantly knocks down the mass of USP6-induced tumors, whether completely or partially.

Liu and Wen et al. found that USP6 levels were elevated in bone marrow aspiration specimens from CML patients, which was associated with a poor prognosis. USP6 expression was considerably higher in imatinib (IM)-resistant clinical samples compared to IM-sensitive samples. Overexpressing USP6 significantly decreased IM-induced apoptosis in leukemia cells. Overexpressing USP6 led to increased GLS1 ubiquitination and decreased GLS protein [52]. A mechanistic research found that USP6 regulates IM resistance in CML cells via GLS1 and miR-146a-5p. The literature revealed that miR-146a-5p is downregulated in patients with IM-resistant CML and plays a role in signaling DNA damage and activating cell cycle checkpoints (USP6). Bioinformatic study of USP6 revealed a possible binding site for miR-146a-5p in its 3′UTR. A mutation in USP6’s miR-146a-5p-binding region inhibited miR-146a-5p’s action on the USP6 3′UTR. The study found that miR-145-5p suppressed USP6 by binding to its 3′UTR. They analyzed miR-146a-5p levels in IM-resistant and IM-sensitive cells and clinical samples from cohort 1. qPCR results showed a substantial drop in miR-146a-5p in IM-resistant cells. MiR-146a-5p levels were significantly lower in IM-sensitive patients than in normal controls and even lower in IM-resistant patients. Pearson’s correlation analysis revealed a negative association between miR-146a-5p and USP6. Next, cells were transfected with miR-146a-5p mimics, and USP6 was overexpressed. Transfecting miR-146a-5p mimics decreased glutamine uptake and increased IM-induced cell death, which was reversed by overexpressing USP6. This study found that miR-146a-5p effectively targeted USP6.

MicroRNA-130a (miRNAs or miRs) are a type of micro-RNA molecules that range in length from 21 to 25 nucleotides and play significant roles in a number of biological processes, including cell proliferation, migration, invasion, and cancer [53, 54]. Zhang et al., aimed to investigate the mechanism whereby miR-130a affects the Wnt/β-catenin signaling pathway by targeting USP6 in Uveal melanoma (UM) [55] Bioinformatics study and a dual-luciferase reporter gene test revealed an interaction between miR-130a and USP6. Gain- and loss-of-function experiments of miR-130a and USP6 were conducted in nude mice to assess UM cell motility, invasion, and tumor formation. miR-130a expression decreased in uveal tissues from UM patients, particularly in metastatic uveal tissues. UM patients with low miR-130a expression had a decreased overall survival rate compared to those with strong expression. Overexpressing miR-130a or inhibiting USP6 in UM MUM-2B and MUM-2C cell lines reduced Wnt, β-catenin, and EGFR expression, activated SMAD4 expression, and reduced UM cell motility and invasion in vitro [21]. These studies proved to involve various cellular processes to knock down USP6 and hinder the growth of tumor cells as well as disease progression. To further elucidate the suppression of USP6, a number of inhibitors have been reported, specified as follows.

## Diseases Associated with USP6

**Fig. 4:**
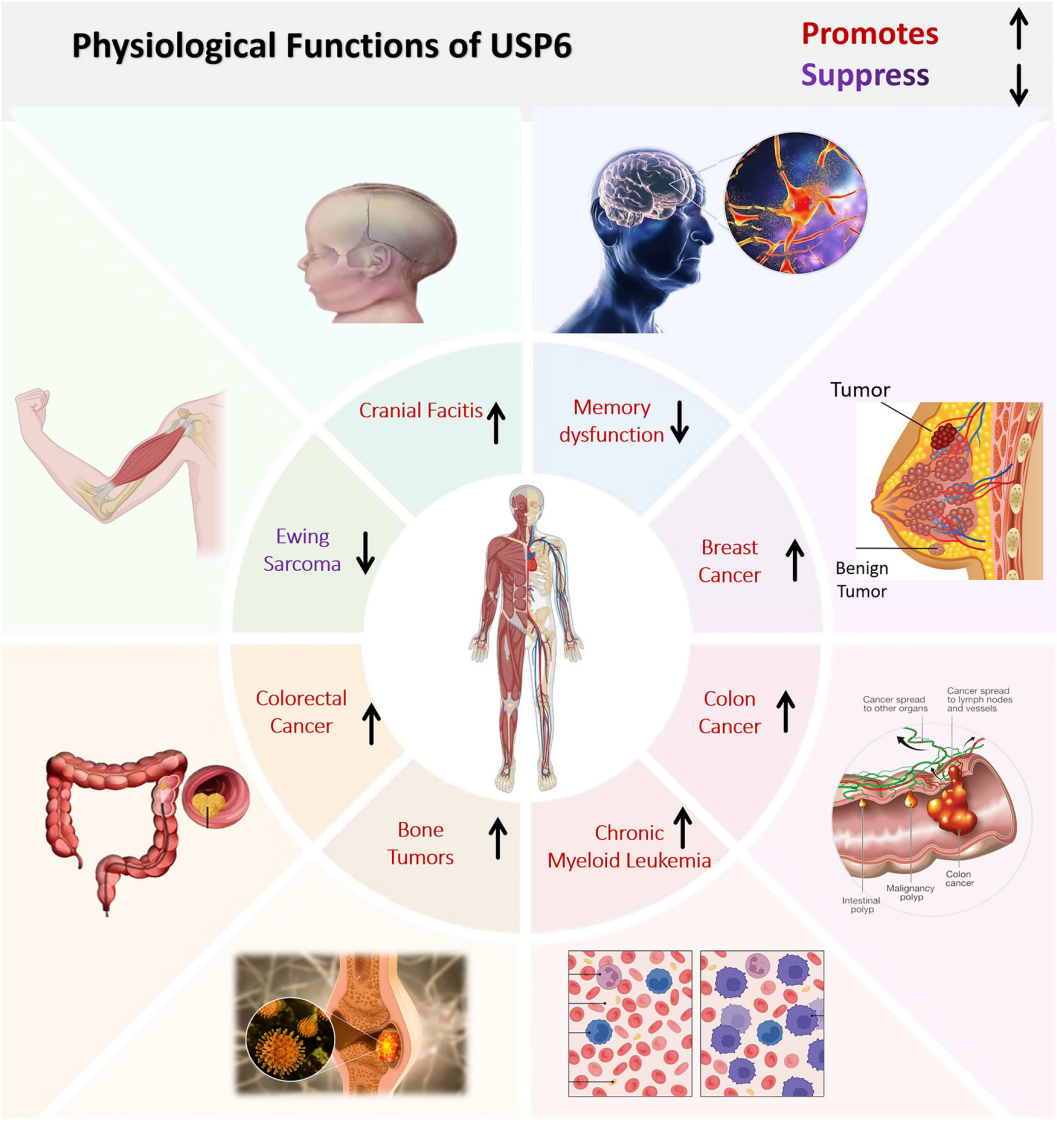
Targeting USP6 associated with bone tumors, Cranial fasciitis, breast cancer, colon cancer, colorectal cancer, and memory dysfunction.

Aberrant expression of USP6 is implicated in the development of a diverse array of diseases, including bone tumor, cranial fasciitis, breast cancer, memory dysfunction, colon and colorectal cancer, summarized in Fig. 4 and Table 1.

**Table 1:** Represents diseases associated to USP6 dysregulation.

Disease	Protein (USP6)	Target	Mechanism	Reference
Aneurysmal bone cyst	Overexpressed	TRAF6, c-junction	Overexpressed USP6 bind with TRAF6, and c-junction to regulate aneurysmal bone cyst.	[52]
Nodular fasciitis	Overexpressed	TRAF6, c-junction collagen 1	Overexpressed USP6 bind with TRAF6, and c-junction, and collagen 1 and cause nodular fasciitis.	[53, 54]
Cranial fasciitis	Overexpressed	Wnt pathway	USP6 as an oncogene promotes cancer cell growth, invasion, by regulating Wnt pathway.	[58]
Breast cancer	Overexpressed	transforming growth factor beta	USP6 regulate transforming growth factor beta (TGFβ) signaling, has a well-documented role in mediating epithelial-to-mesenchymal transition (EMT), tumor progression and metastasis in breast cancer.	[64]
Colon cancer	Overexpressed	GOLPH3 and COAD	Overexpression of USP6 promoted COAD cell viability, inhibited apoptosis, and accelerated the growth of transplanted tumors growth in vitro and in vivo by deubiquitinating GOLPH3	[69]
Colorectal cancer	Overexpressed	Wnt/β-catenin pathway	USP6 with high expression in CRC tissues regulates CRC cell proliferation	[73]
Memory dysfunction	Overexpressed	NMDAR, GluN1	USP6 SP6 enhances GluN1. NMDAR stability to promote synaptic function and cognition	[79]
Chronic myeloid leukemia	Overexpressed	GLS1	Overexpression of USP6 reduces GLS1 ubiquitination, preventing breakdown and allowing for continued glutamine metabolism. This metabolic adaptation leads to CML cells’ resistance to imatinib-induced apoptosis	[70, 71]

### USP6 as a tumor promoter

Ubiquitin-Specific Protease 6 (USP6) exhibits a dual role functioning both as a tumor promoter and a tumor suppressor. USP6 expression and activity have been implicated in the development and progression of various human malignancies, underscoring its potential as a therapeutic target. When USP6 is overexpressed due to genetic rearrangements or fusions, contributes to tumor formation. Its ability to stabilize c-Jun (a proto-oncogene and transcription factor) enhances c-Jun-dependent signaling pathways that promote cell proliferation and invasion, thereby facilitating tumorigenesis. The interaction between USP6 and c-Jun contributes to the development of various tumors, including benign mesenchymal neoplasms like nodular fasciitis and aneurysmal bone cysts.

One of the most striking features of USP6 in the context of cancer is its involvement in chromosomal translocations. These translocations result in the fusion of the USP6 gene with other gene partners, leading to its overexpression. The overactive USP6 protein promotes cell survival, proliferation, and resistance to apoptosis, all of which are significant features of cancer growth. The anomalous expression of USP6, often driven by these translocations, has been implicated in various types of tumors, particularly in soft tissue sarcomas and other malignancies, where it accelerates tumorigenesis by disrupting normal cellular processes.

#### USP6 in bone tumors

A wide expression of USP6 is seen in several human cancer types. The initial description of USP6 rearrangement was published more than 15 years ago in an aneurysmal bone cyst (ABC), which is thought to be a benign bone tumor. Afterwards, a number of USP6 fusion partners have been identified, all of which cause USP6 to become transcriptionally activated by juxtaposing this gene with an ectopic promotor. USP6 activates several pathways essential for the development of tumors. Recurrent USP6 rearrangement was discovered by Oliveira and colleagues in 2004 in primary aneurysmal bone cysts (ABCs) (63%) [56]. According to recent research, over 60% of ABC instances involve translocation of the USP6 locus, which results in USP6 overexpression. At least a subset of ABCs is thought to harbor USP6 translocation, which is thought to be carried by immature osteoblasts. The most frequent fusion partner for USP6 in primary ABC was found to be CDH11. Furthermore, a recent study discovered that USP6 binds to TNF receptor-associated factor 6 (TRAF6) at the c-junction to induce ABC. In nodular fasciitis (NF), recurrent USP6 rearrangement and the most frequent fusion partner, MYH9, were identified. Between 74 and 100% of nodular fasciitis cases at any anatomic location have been documented to have USP6 gene rearrangements (A). Following the identification of USP6 fusions in nodular fasciitis in 2011, 16 cases involving the breast or axilla have been documented, with TRAF6, c-jun, and collagen 1 being the targets [57–60].

In recent years, growing numbers of molecular genetic investigations have increased the families of neoplasms related to USP6 [37, 61]. The family also comprises the following conditions: spindle cell neoplastic lesions: benign infiltrative myofibroblastic neoplasms, fibro-osseous pseudotumor of digits (FOPD), fibroma of tendon sheath (FTS), soft tissue aneurysmal bone cyst (ST-ABC), and myositis ossificans (MO) [62]. Variants of NF include fasciitis ossificans (FO), intravascular fasciitis (IVF), and cranial fasciitis (CF) [63]. Notably, proliferative myofibroblasts/fibroblasts with or without osteoid component metaplasia are characteristics of USP6-associated neoplasms [64]. Numerous tumors in this family are typical pseudosarcomatoid lesions, which can be difficult to distinguish from cancers during the diagnostic process, particularly in cases where the tumors include metaplasia of the bone [23, 25–27, 62, 65]. The most common soft tissue tumors associated with USP6 and bone metaplasia include FO, FOPD, MO, and ST-ABC. From a morphological standpoint, MO and FOPD have long been identified as tumors that belong to the same spectrum [47]. Recent research showed that the most common fusion partner in MO and FOPD is COL1A1, which differs from the primary ABC and NF.

While the 2020 WHO classification of soft tissue and bone tumors and some recent researchers have suggested that rare ST-ABC, MO, and FOPD should be classified into the same subclass of USP6-associated tumors. Genetic changes and clinicopathological features similar to MO/FOPD have recently been identified in ST-ABC. Notably, FO also had ossifying components in its morphology; nevertheless, the limited studies that have been done on these tumors with small sample sizes are noteworthy. More research is required to ascertain whether FO, MO, FOPD, and ST-ABC have a closer affinity [66].

#### Cranial fasciitis

It is a benign myo fibroproliferative lesion that usually affects children and affects the scalp and underlying bones [67]. It shares histological characteristics with the closely related variation of nodular fasciitis, including loose fascicles of stellate cells on a fibromyxoid background. They were formerly classified as a reactive process, nodular fasciitis patients were reclassified as clonal neoplastic processes with the discovery of USP6 translocations in over 90% of cases. A recent study performed experiments in which fifteen archive examples of cranial fasciitis, consisting of fresh frozen tissues and formalin-fixed paraffin-embedded tissues. Samples were assessed using an RNA-based targeted sequencing panel that targeted genes, such as USP6, frequently altered in neoplasia. Three of the fifteen instances (five out of fifteen) tested positive for USP6 rearrangements anticipated to fuse the whole USP6 coding region to the 5′ partner’s promoter. These three new cases included two new cases of Serpinh1-USP6 and one each of Col3A1-USP6, SPARC-USP6, and MYH9-USP6. These findings show that USP6 rearrangements in cranial fasciitis are recurring and emphasize how well-targeted RNA sequencing works to find known and unknown fusion partners. Understanding the fundamental biology of cranial fasciitis is aided by the discovery of USP6 promoter-swapping rearrangements, which also strengthen the biological connection between the condition and nodular fasciitis. The number of benign fibro-osseous lesions linked to USP6 gene changes is expanding with the finding of USP6 involvement in CF. This discovery demonstrates a genetic connection between CF and other tumors with altered USP6. Understanding the role of USP6 gene rearrangements in cranial fasciitis is a major step forward in treating this uncommon pediatric tumor. In addition, it emphasizes the significance of molecular pathology in pediatric soft tissue neoplasms and improves diagnostic techniques while placing CF into the larger framework of USP6-associated tumors [63, 68].

#### USP6 in breast cancer

One significant finding from the USPs linked to breast cancer is that they are essential modulators of transforming growth factor beta (TGFβ) signaling, which is known to play a role in mediating the breast cancer’s epithelial-to-mesenchymal transition (EMT), tumor growth, and metastasis. According to a recent study, USP6 also contributes to resistance to hormone treatment and chemotherapy by improving the invasion and migratory capacities of breast cancer. In a subpopulation of aggressive basal-like breast tumors, USP6 overexpression increases glycolysis in breast cancer cells and points to a metabolic vulnerability that can be targeted with certain treatment drugs [69]. Research suggests that USP6 may facilitate breast cancer spread by stabilizing proteins like matrix metalloproteinases (MMPs) that increase cancer cellṣ’ ability to invade. As phenotypic discovery progresses, more information on USP6 and its inhibitors is needed, which will lead to the identification of more effective and focused breast treatment candidates [70].

#### USP6 in colon cancer

USP6 is substantially expressed in colon cancer tissues at the mRNA and protein levels. Elevated expression of USP6 was directly linked to colon cancer invasion and metastasis. Both in vitro and in vivo colon cancer cell invasion and metastasis are facilitated by the Overexpression of USP6. It is thought that USP6 is a new gene that facilitates colon cancer invasion and metastasis.

Zheng et al. use immunohistochemical staining analysis, western blot, and RT-PCR to assess the USP6 at the mRNA and protein levels in colon cancer patients. The findings showed that poor disease-specific survival and overall survival were predicted by using Kaplan-Meier analyses with log-rank tests, univariate and multivariate Cox analyses, and high USP6 expression. Additionally, a cell function study showed that USP6 could encourage the in vivo invasion of colon cancer cells and their liver metastases. These results suggested that elevated USP6 expression aided in the development of colon cancer and suggested that USP6 might be a useful predictor of prognosis for colon cancer patients [71]. In the chemoresistance of colon adenocarcinoma (COAD), USP6 directly controlled the GOLPH3 protein’s deubiquitination and improved its stability. By deubiquitinating GOLPH3, Overexpression of USP6 increased the viability of COAD cells, prevented apoptosis, and sped up the growth of transplanted tumors both in vivo and in vitro. Furthermore, DDP-resistant colon cancer cells exhibited high expression levels of circCYFIP2, which encouraged cell proliferation. Through mechanical binding, circCYFIP2 enhances the interaction between GOLPH3 and USP6, inducing DDP resistance both in vitro and in vivo.

To sum up, USP6 functions as a deubiquitinase that targets and increases the stability of the GOLPH3 protein in COAD. In the meantime, circCYFIP2 serves as a scaffold to impart platinum resistance and is essential for the deubiquitination of GOLPH3 protein mediated by USP6. Finding the circCYFIP2/USP6/GOLPH3 route is a viable avenue to address COAD chemoresistance [72, 73].

#### USP6 in colorectal cancer

Among different types of cancer, colorectal cancer (CRC) is classified as the third most commonly reported cancer after breast and lung cancers. It is ranked as the second most common cause of cancer death [1]. The majority of people with colorectal cancer (CRC) have a poor prognosis and are discovered at an advanced or metastatic stage. Through the Wnt/β-catenin pathway, USP6, which is highly expressed in CRC tissues, controls the proliferation of CRC cells. USP6 has an impact on CRC growth. Research by Kang et al. revealed that USP6 was elevated in CRC patients’ tumorous tissues. In human CRC cell lines, USP6 knockdown reduced cell proliferation, caused G0/G1 cell cycle arrest, and stopped cells from becoming tumorigenic in nude mice. This was linked to the suppression of the Wnt/β-catenin pathway. Furthermore, β-catenin’s ubiquitination increased with USP6 knockdown, suggesting that USP6 functioned as a deubiquitinase to control the accumulation of β-catenin [74].

#### USP6 in Chronic Myeloid Leukemia

Chronic Myeloid Leukemia (CML) is a white blood cell cancer distinguished by the uncontrolled proliferation of myeloid cells at various stages of maturity. It is primarily caused by the BCR-ABL fusion gene, which results from a chromosomal rearrangement known as the Philadelphia chromosome. This fusion gene generates a constitutively active tyrosine kinase, which promotes uncontrolled cell proliferation while inhibiting apoptosis, resulting in the buildup of immature myeloid cells. USP6 has been discovered as a crucial role in CML, especially in terms of treatment resistance [75]. Studies have shown that USP6 suppresses GLS1 ubiquitination, resulting in an increase in GLS1 protein levels. This relationship is especially important in imatinib resistance, as USP6 is increased in resistant CML cells compared to sensitive ones. Overexpression of USP6 reduces GLS1 ubiquitination, preventing breakdown and allowing for continued glutamine metabolism [52]. This metabolic adaptation promotes cell survival and proliferation, leading to CML cells’ resistance to imatinib-induced apoptosis. Additionally, miR-146a-5p mediates the control of USP6 on GLS1, indicating a complicated interaction between these molecules that promotes chemoresistance [76]. Therefore, USP6 is a viable therapeutic target for overcoming resistance in CML treatment because it inhibits GLS1 ubiquitination, which not only improves GLS1 stability but also plays a crucial role in the survival of CML cells under therapeutic stress.

### USP6 as a tumor suppressor

The contrasting roles of USP6 as both a tumor suppressor and promoter underscore the complexity of its function in cancer biology. It can modulate immune responses while also participating in oncogenic processes. USP6 enhances the immune response against tumors, creating an immunostimulatory microenvironment important for effective anti-tumor activity. This duality is particularly evident in its involvement with Ewing Sarcoma (ES), a challenging pediatric bone cancer [77]. USP6 exhibits tumor-suppressive functions in Ewing sarcoma by enhancing immune activation and chemokine production (such as CXCL10 and CCL5), which could counteract tumor growth. USP6 works by directly deubiquitinating JAK1, which stabilizes and activates it. The STAT1 and STAT3 transcription factors are then phosphorylated as a result. In addition to making ES cells hypersensitive to exogenous Type I and II IFNs, as evidenced by increased and prolonged STAT1 phosphorylation and synergistic induction of IFN-stimulated genes (ISGs), USP6 is sufficient to initiate an IFN response gene signature in both ES cells and other neoplastic models. It is yet unknown, though, how USP6 and its IFN signaling activation affect ES pathogenesis. In fact, depending on the cancer environment, IFNs can have either pro- or anti-tumorigenic actions.

### USP6 suppresses memory dysfunction

USP6 exhibits significant expression in induced human neurons, and neuron-specific expression of USP6 improves learning and memory in a transgenic mice model. In USP6 transgenic mouse hippocampus, USP6 expression controls long-term depression and long-term potentiation that are dependent on the N-methyl-D-aspartate-type glutamate receptor (NMDAR). The transgenic USP6 mouse cortex’s proteome analysis showed reduced NMDAR ubiquitination increased NMDAR expression, stability, and cell surface distribution when USP6 is overexpressed. In transfected cells, USP6 positively regulates GluN1 expression in neurons generated from human embryonic stem cells. Downregulation of USP6 hinders focused GluN1 distribution at postsynaptic density and compromises synaptic function. The findings suggest that USP6 promotes synaptic function and cognition by stabilizing NMDAR [78].

## USP6 inhibitors

**Fig. 5:**
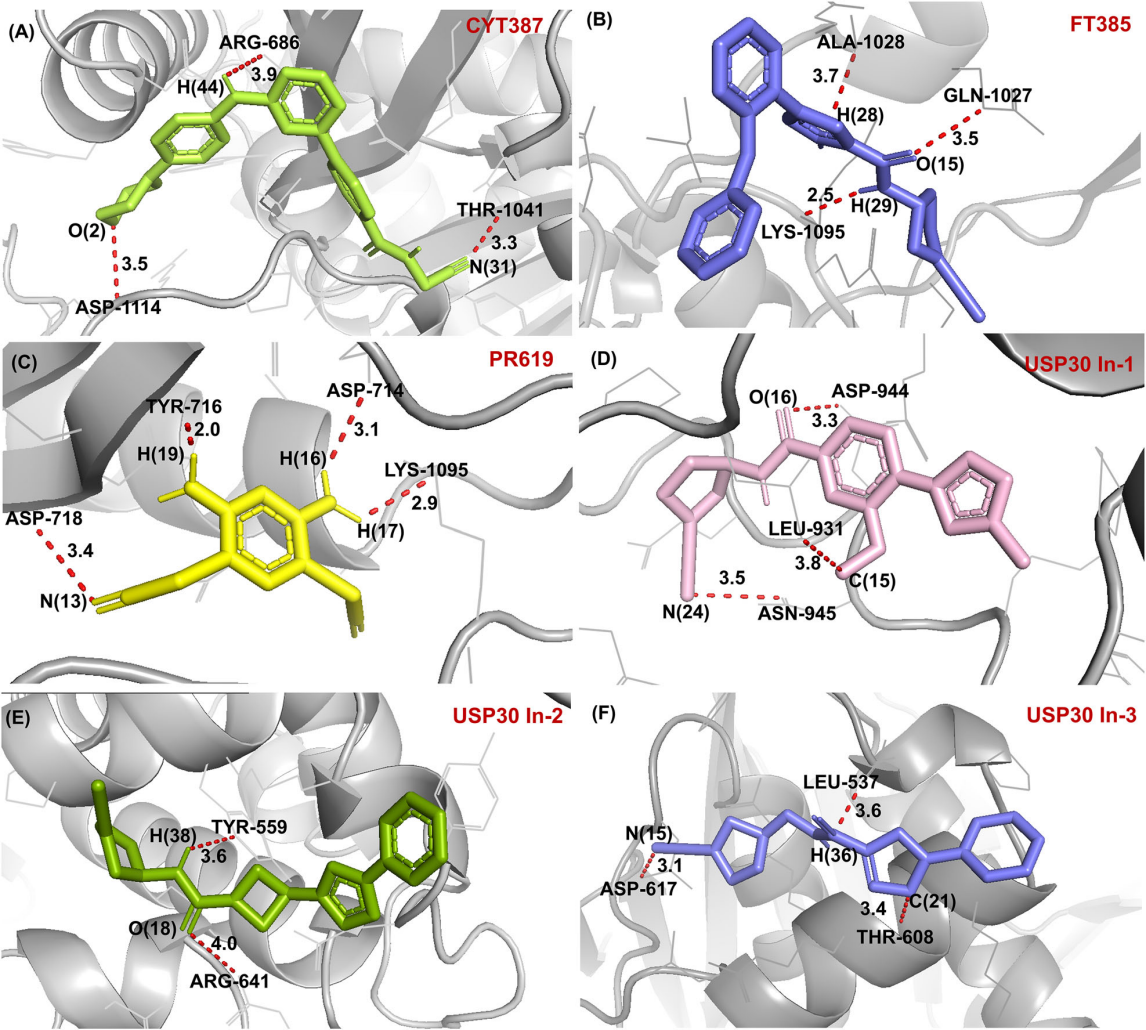
Simulated Molecular Interactions of USP6 with Selected Inhibitors, and closeup views of the compound binding site highlighting key residues. **A** USP6–CYT387, (**B**) USP6-FT385, (**C**) USP6-PR619, (**D**) USP6-USP30 In-1, (**E**) USP6-USP30 In-2, (**F**) USP6-USP30 In-3.

Various USP6 inhibitors such as Momelotinib (CYT387), FT385, USP30 Inh-1, -2 and -3, 2,6-Diaminopyridine-3,5-bis(thiocyanate) (PR-619), and miR-130a were identified against tumor cancers, uveal melanoma, and osteosarcoma[21]. These inhibitors play a significant role in reducing the risk of such diseases. Here, we summarized USP6 inhibitors (Fig. 5). Emerging research has revealed novel physiological roles for USP6 in cancers and other diseases. Mechanistic insights now point to the significant therapeutic potential of USP6 inhibition, positioning it as a promising target for advanced therapeutic strategies. The development of inhibitors for USP6 holds significant therapeutic potential due to the enzyme’s critical roles in various biological processes and disease mechanisms. The selected inhibitors, discussed in the following section, were docked with the USP domain of USP6, as illustrated in Fig. 5. The structural model of USP6 used for simulations was predicted using AlphaFold 2, while the inhibitors were designed and optimized in PyMOL. Molecular docking was performed using AutoDock Vina software.

### Momelotinib (CYT387)

Clinical approval has been granted for momelotinib (CYT387), a selective small-molecule inhibitor of JAK1/2, to treat myelofibrosis [79, 80]. Laura et al. recently investigated the potential of a JAK family inhibitor to stop USP6 from forming tumors. They employed the in vivo effective pan-JAK inhibitor CYT387 for these investigations. Mice were given subcutaneous injections of USP6 cells. Two cohorts of animals received therapy with CYT387 twice a day, whereas the other group received vehicle treatment. Large, highly vascularized tumors were seen in the control group after three weeks, as was previously described.

On the other hand, tumors taken from animals treated with CYT387 simultaneously showed a considerable reduction in size and avascularity. All of these findings suggest that JAK-STAT3 is activated in neoplasms with USP6 translocation and that focusing on this pathway could be a useful strategy for reducing the growth of tumors caused by overexpression of USP6 [81]. From the simulated docked data of USP6 and CYT387 in Fig. 5A, a clear interaction between the protein and ligand is observed. The yellow dots indicate hydrogen bonding between the inhibitor CYT387 and the side chain of ARG-686 in USP6. The bond distance of 3.9 Å suggests a moderately strong hydrogen bond, contributing to the stabilization of CYT387 within the active site. This interaction is important for anchoring the inhibitor in the binding pocket and improving binding specificity and ultimately hindering the active site.

Another hydrogen bond between CYT387 and the carboxyl group of ASP-1114 with a distance of 3.5 Å is displayed also results in stabilizing the inhibitor. By blocking the active site, CYT387 disrupts the JAK-STAT pathway, halting downstream signaling that promotes tumor growth and survival.

### FT385

FT385, a covalent inhibitor of the USP30 deubiquitinating enzyme with a cyanopyrrolidine warhead, was described by Rusilowicz-Jones et al. [82–84]. It has been difficult to find specific active-site inhibitors of deubiquitinating enzymes; nonetheless, FT385 currently offers a useful tool for researching the biology of USPs. When FT385 was evaluated against other DUBs, it solely inhibited USP6 at 200 nM in the case of cancer cells, indicating great selectivity in inhibiting the cleavage of Ub-Rho110 at 1 nM IC_50_ [85]. The molecular interactions between the USP6 protein and the FT385 inhibitor with key focus areas marked by yellow dashed lines can be seen in Fig. 5B. FT385 forms a hydrogen bond with the ALA-1028 at a distance of 3.7 Å, indicating a moderate-strength interaction. The carbonyl oxygen (O(15)) of FT385 acts as a hydrogen bond acceptor and interact with the amide group of GLN-1027 with a bond distance of 3.5 Å. Additionally, a strong hydrogen bond is formed between a hydrogen atom (H(29)) of FT385 and LYS-1095, with a bond distance of 2.5 Å. These interactions primarily stabilize FT385 within the USP6 binding pocket, potentially inhibiting its enzymatic activity.

### 2,6-Diaminopyridine-3,5-bis(thiocyanate) (PR-619)

A broad-spectrum deubiquitinating enzyme (DUB) inhibitor, 2,6-Diaminopyridine-3,5-bis(thiocyanate) (PR-619), has been used in cell-based research to examine the function of ubiquitination in several cellular processes. Few data are available for osteosarcoma (OS), despite an increasing number of research studies highlighting the function of ubiquitin-specific proteases (USPs) in the development of various malignancies. In this context, elevated expression of four USPs (USP6, USP27x, USP41, and USP43) in OS tumor cells has been revealed by RNA-sequencing investigation of OS cells and mesenchymal stem cells that have developed into osteoblasts or not. These four USPs exhibit protein-level nucleic and/or cytoplasmic expression, as shown by tissue microarray examination of patient biopsies.

According to Kaplan-Meyer analysis, it’s interesting to note that patient survival is connected with the expression of two USPs, USP6 and USP41. Ultimately, it is shown through in vivo studies PR619 increases protein ubiquitination in OS cell lines and inhibits the growth of primary OS tumors and the formation of lung metastases. In this regard, in vitro tests demonstrate that PR619 reduces OS cell viability mostly through triggering a caspase3/7-dependent cell death [86]. In Fig. 5C, PR619 interacts with the peptide backbone of USP6 through non-covalent polar contacts, aligning its functional groups with catalytic residues. The distances between PR619 and interacting residues range from 2.0–3.5 Å, indicative of strong and stable binding. Notably, the close proximity of LYS-1095, ASP-718, TYR-716, and ASP-714 interactions reflects the inhibitor’s ability to block USP6 activity effectively. The aromatic ring in the inhibitor aligns closely with a planar residue contributing to binding through π-π interactions. However, this specific interaction is not explicitly highlighted. PR619 stabilizes the USP6 in an inactive conformation, likely preventing substrate access to the catalytic cysteine. The central aromatic ring in the structure of PR619 engages hydrophobic residues, offering dual hydrophobic and polar bonding underscores the potential of PR619 as a therapeutic agent targeting USP6.

### USP30 Inh-1, -2 and -3

Based on the published WO201710614 novel compound structures, three structurally related small molecule USP30 inhibitors (which refer to as USP30 Inh-1, -2, and -3) were synthesized. Using recombinant USP30 and the fluorogenic artificial DUB substrate ubiquitin-rhodamine 110 (Ub-Rho110), the inhibitory action of USP30 was evaluated biochemically. With estimated IC_50_ values of 15–30 nM, USP30Inh-1, -2, and -3 all strongly inhibited USP30-mediated cleavage of Ub-Rho110. USP30 inhibitor selectivity was evaluated using the Ubiquigent DUB profiler™ service. At 1 µM, USP30Inh-1, -2, and -3 showed strong selectivity against more than 40 known DUB enzymes. Although these inhibitors are not USP6-specific but can inhibit USP6 at higher concentrations of around 10 µM, all compounds showed decreased selectivity, with USP6 showing the highest off-target inhibition [87].

The molecular docked structure of USP6 and USP30 Inh-1 Fig. 5D shows the spatial arrangement of the inhibitor suggests its ability to fit tightly into the active site of USP6. The distances between atoms (as indicated, from 3.3–3.8 Å) are within the typical range for non-covalent interactions like hydrogen bonds or van der Waals forces. A hydrogen bond is observed between the inhibitor and ASN-945 residue of USP6, lone pair electrons from the oxygen of UNK-1 shows binding with ASP-944 and the inhibitor appears to be making hydrophobic interactions with residues LEU-931. The simulated binding interactions suggest that these residues might contribute to the binding affinity and specificity through hydrophobic contacts.

The binding interaction of USP6 with the USP30 Inh-2 inhibitor, as shown in Fig. 5E, highlights the molecular details of inhibition in the catalytic domain. The inhibitor USP30-In2 forms a hydrogen bond with the hydroxyl group of TYR-559. In addition, the ARG-641 side chain interacts with USP30-In2, possibly via electrostatic interactions. The orientation of the ligand within the protein’s binding pocket suggests that it is stabilized by the additional π-π interactions of aromatic rings present in both structures. Unlike other inhibitors that might induce large conformational shifts (e.g., Phe rotation in USP7), USP30-In2 primarily stabilizes the existing conformation of USP6.

Figure 5F displays the structure of USP6 in complex with the inhibitor USP30 In-3 reveals that the carbonyl group of USP30-In3 forms a hydrogen bond with ASP-617, stabilizing the inhibitor in the binding pocket. The interaction distance of 3.1 Å reflects strong hydrogen bonding and enhances binding affinity. Furthermore, USP30 In-3 interacts with THR-607 through van der Waals interactions and improves the stability of the inhibitor’s position. The inhibitor likely leverages this residue’s flexible side chain for proper alignment in the active site. USP30 In-3 also occupies a hydrophobic pocket formed by LEU-637, demonstrating the stability of the inactive conformation of USP6. The binding of USP30 In-3 likely locks USP6 in an inactive state, reducing its enzymatic activity. The molecular interactions highlight its potential for selective inhibition of USP6 and provides a structural basis for future inhibitor optimization.

## Conclusion and future perspective

This review highlights the importance of USP6 as a pivotal regulator of various cellular processes, influencing key signaling pathways such as NFκB, JAK-STAT, and Wnt, which are integral to cellular homeostasis, inflammation, and cancer progression. A 3-D structure of USP6 and its full-length human sequence are predicted to elucidate the structural features. In addition, the detailed discussion on the diseases involved USP6, such as bone cancer, breast cancer, colon cancer, and neurodegenerative diseases, and the identification of potential inhibitors marks an exciting step toward interpreting this knowledge into clinical applications. However, significant challenges remain in the research and development of USP6-targeted therapies. First, the complexity of USP6’s diverse functions necessitates a comprehensive understanding of its molecular mechanisms across different tissue types. This complexity is further compounded by the fact that the 3-D molecular structure of USP6 is yet to be discovered.

Additionally, the development of highly selective USP6 inhibitors with minimal off-target effects is a major hurdle. Looking to future research aims, USP6 holds promise not only for cancer therapy but also for the treatment of neurodegenerative diseases and disorders. The growing field of structural biology surrounding the role of USP6 paves the way for novel and targeted therapies. Future studies will need to focus on refining our understanding of the regulatory mechanisms of USP6, developing more specific and effective inhibitors, and investigating the therapeutic potential of USP6 modulation in clinical settings.
